# Deciphering Resting Microglial Morphology and Process Motility from a Synaptic Prospect

**DOI:** 10.3389/fnint.2015.00073

**Published:** 2016-01-19

**Authors:** Ines Hristovska, Olivier Pascual

**Affiliations:** ^1^INSERM U1028, CNRS UMR5292, Lyon Neuroscience Research CenterLyon, France; ^2^Université Claude Bernard Lyon 1Lyon, France

**Keywords:** microglia, motility, neuronal activity, ATP, glutamate

## Abstract

Microglia, the resident immune cells of the central nervous system (CNS), were traditionally believed to be set into action only in case of injury or disease. Accordingly, microglia were assumed to be inactive or resting in the healthy brain. However, recent studies revealed that microglia carry out active tissue sampling in the intact brain by extending and retracting their ramified processes while periodically contacting synapses. Microglial morphology and motility as well as the frequency and duration of physical contacts with synaptic elements were found to be modulated by neuronal activity, sensory experience and neurotransmission; however findings have not been straightforward. Microglial cells are the most morphologically plastic element of the CNS. This unique feature confers them the possibility to locally sense activity, and to respond adequately by establishing synaptic contacts to regulate synaptic inputs by the secretion of signaling molecules. Indeed, microglial cells can hold new roles as critical players in maintaining brain homeostasis and regulating synaptic number, maturation and plasticity. For this reason, a better characterization of microglial cells and cues mediating neuron-to-microglia communication under physiological conditions may help advance our understanding of the microglial behavior and its regulation in the healthy brain. This review highlights recent findings on the instructive role of neuronal activity on microglial motility and microglia-synapse interactions, focusing on the main transmitters involved in this communication and including newly described communication at the tripartite synapse.

## Introduction

Microglia are the resident immunocompetent cells of the central nervous system (CNS) and they comprise around 5–12% of the glial cell population (Gomez-Nicola and Perry, 2015). Microglia emerge from two sources: erythromyeloid precursors of the embryonic yolk sac, and myeloid progenitors that invade the CNS and proliferate during embryonic and postnatal development (Ginhoux et al., 2013).

**Figure 1:**
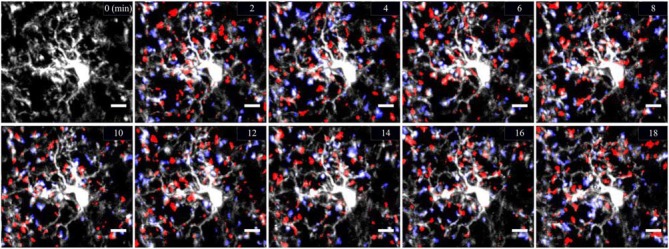
**Cortical two-photon imaging on awake mouse.** Resting microglia are highly ramified and motile in the mouse cerebral cortex. Microglia are labeled by enhanced green fluorescent protein (eGFP) expressed under the control of microglial promoter CX3CR1. Microglial motility is shown by means of representative color-coded time-lapse images of a single microglial cell showing rapid process extensions (in blue) and retractions (in red) with a 2 min interval over the time course of 18 min. Scale bar = 10 μm.

Considered as the resident immune cells of the brain, microglia have been mostly studied in immune and inflammatory contexts (Prinz and Priller, 2014). However, recent *in vivo* data indicate that under physiological conditions, microglial cells exhibit a highly ramified morphology characterized by motile processes that constantly monitor their immediate surrounding by extending and retracting their processes (Davalos et al., 2005; Nimmerjahn et al., 2005). This constant movement of microglial processes while the soma remains stationary is called microglial motility (Figure 1). The unexpected finding of microglial process motility led scientists to enquire and identify new roles in the non-pathological brain (Kettenmann et al., 2013). Since then, microglia were shown to be involved in the phagocytosis of synaptic elements during the entire lifespan, and the formation of learning-dependent synapses in the mature brain, as well as maturation and plasticity of excitatory synapses (Tremblay et al., 2010; Paolicelli et al., 2011; Hoshiko et al., 2012; Schafer et al., 2012; Parkhurst et al., 2013). These functions require localized fine-tuning involving specialized cellular function to deliver targeted messages at individual synapses. This targeted delivery could be compatible with the rapid process motility described earlier. A growing body of evidence also suggests that process motility and the frequency and duration of physical contacts with synaptic elements are regulated by neuronal activity, sensory experience and neurotransmission. It is thus crucial to better understand the mechanisms guiding neuron-to-microglia communication to further comprehend microglial functions in a healthy brain. In this review, we synthetize the recent discoveries on the properties of microglia in physiological conditions. Then, we report findings on the instructive role of neuronal activity on microglial motility and microglia-synapse interactions. Finally, we describe the current understanding of the molecular mechanisms underlying these interactions.

## Microglia, Highly Motile Cells That Contact Synapses

Until fairly recently, due to lack of proper tools to study the microglial cells in a healthy brain, these cells were believed to be immunologically quiescent and were qualified as resting/dormant under physiological conditions. Two pioneer studies using two-photon microscopy observation of microglial motility in the intact cortical micro-environment reversed the common belief that “resting” microglia in the healthy brain were morphologically static (Davalos et al., 2005; Nimmerjahn et al., 2005). They shifted the concept of microglia from “resting” to **“**surveying” (Hanisch and Kettenmann, 2007). Visualization of microglia was made possible by the development of CX3CR1^GFP^ and Iba1^GFP^ transgenic mice (Jung et al., 2000; Hirasawa et al., 2005). Small-shaped soma of microglial cells remained still overtime, with only 5% moving by 1–2 μm/h (Nimmerjahn et al., 2005). In contrast, microglial processes were morphologically plastic with considerable motility. They presented a similar rate of extension and retraction of around 1.47 μm/min (Nimmerjahn et al., 2005). These findings position microglial cells as the most dynamic CNS cells as no other cells display such morphological plasticity *in vivo*. The dynamism of microglial processes was also confirmed in the mouse spinal cord and retinal explants (Davalos et al., 2005; Lee et al., 2008) and in the zebrafish embryo (Peri and Nüsslein-Volhard, 2008).

These groundbreaking discoveries confirmed the morphological plasticity of microglial ramifications under physiological conditions in distinct CNS structures and species led scientists to envisage a possible contribution in neuronal physiology. Specific, direct and activity-dependent microglia-to-synaptic element contacts in the adult mouse visual and somatosensory cortex *in vivo* were thus demonstrated for the first time (Wake et al., 2009). Using CX3CR1^GFP^/Thy1^YFP^ mice, Tremblay et al. (2010) were able to distinguish microglial cells from neurons in the superficial layers of the developing mouse visual cortex at 4 weeks of age. Intriguingly, microglial processes made physical contacts especially with axon terminals and dendritic spines, but also with perisynaptic astrocytic processes and synaptic clefts. They preferentially localized at the proximity of small, more motile and more frequently eliminated dendritic spines rather than larger dendritic spines. Microglial distal processes enveloped dendritic spines by forming finger-like protrusions (Tremblay et al., 2010). Microglia-neuron contacts were brief and transient, at a frequency of one per hour (Wake et al., 2009), whereas in the developing visual cortex contacts varied in duration between 5 and 30 min (Tremblay et al., 2010). These data suggest that under physiological conditions *in vivo*, microglial processes are highly motile and make direct contacts with synaptic elements at regular frequencies during all stages of life.

## Microglial Motility Is Modulated by Neuronal Activity

The discovery of the incessant dynamism of microglial processes and the transient contacts with synapses under physiological conditions led to new questions: are microglial processes specifically guided by neuronal activity and do they respond to the functional status of synapses? To investigate whether neuronal activity instructs microglial motility and contact with synaptic elements, global excitatory and inhibitory activity were modulated by pharmacological, physiological and genetic means. Initial results were rather negative. Silencing neuronal activity by applying tetrodotoxin (TTX) onto cortical surface *in vivo* had no impact on microglial motility (Nimmerjahn et al., 2005), as well as high frequency stimulation inducing long term potentiation (LTP) in acute hippocampal slices (Wu and Zhuo, 2008). Likewise, reduction of basal activity in the visual cortex *in vivo* by several independent approaches, including binocular eye enucleation, retinal TTX injection and reduction of body temperature, had no effect on basal velocity of microglial processes (Wake et al., 2009). However, with a simultaneous visualization of neurons and microglia, Wake et al. (2009), showed a reduced frequency of microglia-synapse contacts. Since this effect resulted from the aforementioned manipulations of neuronal activity, apart from TTX application, neuronal activity could at least modulate microglia-synapse interaction.

Using physiological approach by modulating sensory experience, Tremblay et al. (2010), studied neuronal activity-dependent microglial behavior in the developing mouse visual cortex. Mice were housed in dark conditions and thus deprived of visual experience during a critical period of development. During deprivation, average sampling and motility of microglial processes were significantly reduced, but the frequency and duration of microglia-synapse contacts remained unchanged. The synaptic target changed: microglia no longer localized next to small spines, but interacted preferentially with larger dendritic spines that subsequently shrank. Re-exposure to daylight restored microglial motility and contact with small dendritic spines.

Microglial motility and activity-dependent interactions with synaptic elements were also studied in the zebrafish optic tectum. By simultaneously monitoring GFP-labeled microglia and levels of spontaneous activity by Ca^2+^ imaging, Li et al. (2012) found that neurons with high level of spontaneous activity steer microglial processes, causing an increased contact frequency between these two elements. Conversely, reducing global activity by overexpressing human inward rectifier K+ channel (Kir2.1) in tectal neurons lowered the likelihood of physical contact (Hua et al., 2005). Visual stimuli increased the total number of bulbous endings, inferring microglia-neuron interaction, which were considerably reduced by TTX application (Li et al., 2012).

Overall, these findings suggest that under physiological conditions the motility of microglial processes and their interaction with synaptic elements can be modulated by neuronal activity.

## Neurotransmitters and Mechanisms Affecting Microglial Motility

The molecular cues which maintain the movement of microglial processes and by which neuronal activity may signal to microglia are under investigation. A variety of classical neurotransmitter and neuromodulator receptors are expressed at the surface of microglia from culture assays, which in turn can cause changes in cytokine release, in membrane potential, cellular morphology and motility (Kettenmann et al., 2011). However, these *in vitro* preparations should be interpreted with caution because microglial phenotype resembles activated state (Ransohoff and Perry, 2009). Recent *in situ* studies indicate that microglia express functional purinergic receptors, whereas local application of other neurotransmitters did not elicit electrical responses, most probably reflecting lack of neurotransmitter receptors (Fontainhas et al., 2011).

## Role of Purines

Adenosine triphosphate (ATP), a neurotransmitter of the CNS, has been identified as the key regulator of microglial morphology and baseline dynamics. Disruption of ATP-dependent signaling in the presence of ATP/ADP hydrolyzing enzyme apyrase decreased the basal motility of microglial processes (Davalos et al., 2005) while application of ATP increased basal motility and cell complexity (Fontainhas et al., 2011). ATP is also involved in directed microglial process outgrowth because focal applications of ATP caused a striking extension of microglial processes towards the source of ATP (Davalos et al., 2005; Dissing-Olesen et al., 2014) and process outgrowth persisted as long as ATP was applied (Dissing-Olesen et al., 2014). In addition, ATP critically mediated microglial process outgrowth towards sites of increased neuronal activity (Li et al., 2012; Eyo et al., 2014). Finally, the extension of microglial processes was found to be propelled by a cell autonomous release of ATP contained in lysosomes, serving ultimately as a motor for motility (Dou et al., 2012).

ATP acts at specific ionotropic (P2X) and metabotropic (P2Y) purinergic receptors that are largely distributed in neurons and glial cells. Initial studies *in vitro* showed that ATP signaling through P2YR induced microglial membrane ruffling (Honda et al., 2001), and that P2YR inhibition, but not P2XR, affected the number and motility of microglial processes towards ablation site (Davalos et al., 2005). The requirement of P2R signaling for neuronal activity oriented motility and formation of bulbous contacts was also observed in the zebrafish optic tectum (Li et al., 2012). A major receptor candidate is P2Y12R, selectively expressed by microglia in the physiological brain. Following ATP release, the extension of microglial processes, but not basal motility, was critically dependent on the activation of P2Y12R, as shown by experiments performed on acute hippocampal slices from P2Y12 KO mice (Haynes et al., 2006; Eyo et al., 2014). P2Y12R accumulated at the tip of microglial processes during ATP-induced process outgrowth, along with Rho GTPase Rac, a key molecule in the cytoskeleton reorganization, whose downregulation abolished oriented microglial process movement in response to neuronal activity (Li et al., 2012; Dissing-Olesen et al., 2014).

Additional factors such as gradient formation and generation of ATP metabolites may be important in mediating motility. ATP is quickly catabolized to other purine molecules by ecto-nucleotidases in the extracellular space (Dunwiddie et al., 1997). Constant release of non-hydrolysable ATP in presence of apyrase was unable to attract microglial processes (Davalos et al., 2005). Furthermore, the contribution of ATP hydrolysis products seem to be critical because blocking ATP hydrolysis using selective ectonucleotidases inhibitor altered microglial outgrowth (Dissing-Olesen et al., 2014). When ATP and metabolites diffuse in the extracellular space and form a chemotactic gradient critical for microglial process outgrowth, their elimination reduced the extent and speed of microglial processes (Davalos et al., 2005). Adenosine seems to be a potential candidate for regulating microglial motility because high levels of adenosine receptors A1 and A3 are expressed on microglia in physiological conditions (Hammarberg et al., 2003) and an interplay of simultaneous purinergic stimulation of both A3 and P2Y12 receptors was found necessary for process outgrowth (Ohsawa et al., 2012). Adenosine was also involved in the retraction of microglial processes in the pathological brain due to signaling involving A2A receptors (Orr et al., 2009).

ATP appears to be released in an activity-dependent manner by neurons and astrocytes through hemichannels (pannexin and connexin), transporters and secretory vesicles (Burnstock, 2008). Studies reviewed below focus on ATP release from hemichannels, since this particular mode of communication has been predominantly investigated in relation to microglial motility. The precise distribution of the three pannexin subtypes (Panx1 to Panx3) between cell types and subcellular location has not been fully understood, but could partly account for discrepancies in the literature (Penuela et al., 2013). Initial studies found that probenecid, a non-selective pannexin channel antagonist, caused a general decrease in morphological parameters and basal velocity (Fontainhas et al., 2011). In the zebrafish optic tectum, glutamate uncaging-induced movement of microglial processes and the formation of bulbous endings were abolished using probenecid and more importantly, by specifically downregulating *pannexin-1* expression while keeping unchanged basal cell area and velocity (Li et al., 2012). Consequently, pannexin-1 emerged as the main hemichannel mediating ATP release and subsequently microglial outgrowth. However, a recent study found that during pannexin-1 blockage and in pannexin-1 deficient mice, microglial outgrowth following NMDAR activation in acute hippocampal slices was unaffected (Dissing-Olesen et al., 2014). This mechanism remained probenecid-dependent, raising possibilities for involvement of other pannexin channels mediating ATP efflux, such as pannexin-2 or prepackaged vesicles (Dissing-Olesen et al., 2014; Eyo et al., 2014).

Another important component affecting microglial dynamics could involve connexin hemichannels that mediate ATP released from astrocytes. They seem to be of particular importance for basal velocity of microglial processes *in vivo* because pharmacological blockade of connexins decreased it significantly (Davalos et al., 2005). However, fluoroacetate, an astrocytic function blocker, did not affect glutamate-induced microglial process extension (Eyo et al., 2014). In acute hippocampal slices, blocking connexin channels did not prevent NMDA-mediated microglial extension (Dissing-Olesen et al., 2014; Eyo et al., 2014). The discrepancy in these findings could be due to tissue specificity, microglia heterogeneity, *in vivo* and *ex vivo* preparation and the type and concentration of the pharmacological substances utilized.

These studies clearly demonstrate that purinergic signaling, in particular ATP and its derivates, are crucial for mediating microglial basal motility and neuronal activity-oriented motility. Further studies need to be performed to address the specific effects and contribution of purinergic molecules, as well as the exact downstream signaling pathways elicited in microglia.

## Role of Glutamate and Gaba

**Figure 2:**
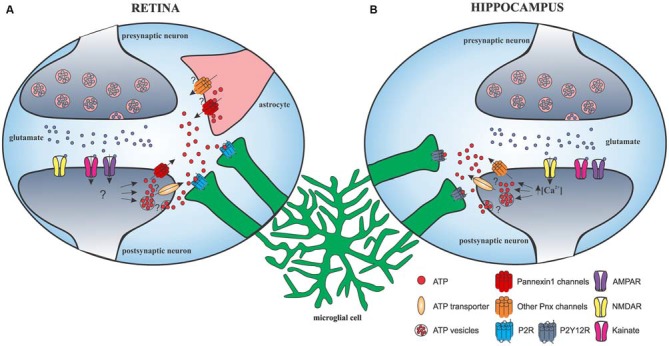
**Schematic representation of glutamatergic neurotransmission-induced microglial process outgrowth in the retina and hippocampus. (A)** In the retina, AMPAR/kainate activation leads to release of ATP through pannexin-1 and possibly other mechanisms from neurons and astrocytes, ultimately leading to microglial response through P2 receptors. **(B)** In the hippocampus, glutamate-induced microglial process outgrowth is dependent on NMDA receptor. NMDAR activation leads to a significant Ca^2+^ influx that is required for ATP release through currently unknown mechanisms but independent of pannexin-1 and astrocyte hemichannels. ATP diffuses into the extracellular space and activates microglial purinergic receptor P2Y12, eliciting microglial process extension.

Direct action of glutamate and γ-aminobutyric acid (GABA) neurotransmission on microglial morphology and motility was also investigated. Initial studies failed to demonstrate any effect of local application of glutamate and GABA on microglial motility. No change was provoked by enhancing neuronal activity with the application of GABA antagonist on the cortical surface (Nimmerjahn et al., 2005) or the application of glutamate and GABA on acute hippocampal slices and spinal cord dorsal horn (Wu and Zhuo, 2008; Chen et al., 2010). A more recent study found that glutamatergic and GABAergic neurotransmission modulated microglial morphology and motility in retinal explants, an *ex vivo* model with minimal CNS damage: GABA application decreased microglial motility, whereas bicuculline increased both the size and basal velocity of microglial processes (Fontainhas et al., 2011). Both agonist and antagonist of ionotropic glutamatergic receptors affected the size and motility of microglial processes mainly through fast AMPA/kainate receptors in retinal explants. This effect was mediated to a much lesser extent through NMDA receptors. Strangely, application of glutamate alone did not result in any change of the above-mentioned parameters (Fontainhas et al., 2011). We must note that variations exist between brain areas. In the hippocampus, two recent studies used acute slices to show an important glutamate-induced microglial process outgrowth requiring NMDAR activation that was independent of AMPA/kainate receptor activation (Dissing-Olesen et al., 2014; Eyo et al., 2014). These differences between structures may attest for striking tissue-specific regulation of microglial motility through fast AMPA/kainate receptors in the retina and slow NMDA receptors in the hippocampus (Figure 2). Glutamate signaling also affected microglial motility in the zebrafish optic tectum. Glutamate uncaging, a noninvasive approach for upregulating neuronal activity, caused outgrowth of microglial processes towards the glutamate source as well as formation of bulbous endings (Li et al., 2012).

Studies have suggested that glutamate and GABA neurotransmission do not signal directly to microglial cells, but affect microglial motility and outgrowth by modulating extracellular levels of nucleotides, such as ATP and metabolites. Local application of glutamatergic or GABAergic agonists did not induce detectable electrophysiological responses in microglia. This is in agreement with the discovery that ionotropic glutamatergic receptors were not present on microglial processes and soma (Fontainhas et al., 2011; Eyo et al., 2014). On the contrary, ATP induced large inward currents in microglia and seems to be indeed implicated downstream glutamatergic transmission because reduction of process length and motility, obtained with glutamate receptor blockade, was inversed by ATP (Fontainhas et al., 2011).

Future studies should investigate whether the activation of all three main ionotropic glutamatergic receptors can activate a common pathway.

## Other Neurotransmitters

Other than glutamate and GABA, very few studies have investigated the impact of neurotransmitter application on microglial motility. Acetylcholine, norepinephrine or serotonin did not cause changes in the dynamism of microglial processes in the spinal dorsal horn (Chen et al., 2010). However, Gyoneva and Traynelis (2013) has shown that norepinephrine was responsible for the retraction of microglial processes in acute brain slices in physiological conditions through β2 adrenergic receptors present in resting microglia. Process extension caused by ATP was inhibited by co-application of norepinephrine. Propranolol, an antagonist of β2 receptor, reversed this effect. These findings raise an interesting question: can signaling mediated by norepinephrine modify the microglial reactivity to ATP release *in vivo*? We believe further studies of the influence of various neurotransmitters in more physiological conditions are needed to obtain a more complete understanding of the mechanisms underlying neuron-to-microglia communication.

## Microglia-Astrocyte Interactions

In addition to interacting structurally with pre- and postsynaptic elements, microglia closely affix perisynaptic astrocytic processes (Tremblay et al., 2010). Accumulating evidence demonstrate that astrocytes not only play an important role in maintaining homeostasis, but also in regulating synaptic transmission (Perea et al., 2014; Oliveira et al., 2015). Thus, it appears that these glial cells may cooperate in the modulation of synaptic function taking into consideration their occasional structural proximity and emerging roles. Despite the possible influence of astrocytic ATP on microglial motility and morphology previously described, astrocyte-derived ATP was also found to induce microvesicle shedding from microglial cells, ultimately generating an increase of excitatory transmission (Bianco et al., 2005; Antonucci et al., 2012). Two recent studies provided compelling data on microglia-to-astrocyte interactions. Pascual et al. (2012) found that LPS stimulation of microglia in acute hippocampal slices resulted in a rapid ATP release. ATP bound to astrocytic P2Y receptor, which led to glutamate release by astrocytes, ultimately increasing excitatory transmission (Pascual et al., 2012). Another study found that fractalkine signaling, mediated exclusively by microglia, caused adenosine release, which in turn increased the release of D-serine most probably from both astrocytes and microglia, finally resulting in potentiation of NMDAR function (Scianni et al., 2013). Future studies must elucidate the context and molecular mechanisms governing interactions between microglia and astrocytes, as well as the functional consequences at the regulation of synaptic transmission and neural circuits.

## Conclusion

Considerable progress has been made to decipher signaling mechanisms that regulate microglia-synapse interactions, which is only a token of its complexity. It is now crucial to better understand these mechanisms because the degree to which and how microglia interact with other cell types is most probably dependent on their morphology and motility. Several studies reveal functional importance of microglia in physiological conditions as they contribute to the fine-tuning of neuronal circuits and engage in synaptic and structural plasticity. These microglial functions are at least partly mediated by their motile processes, which can engulf synaptic terminals, thus homeostatically regulating neuronal activity and secrete a plethora of signaling molecules, including cytokines, neurotrophines, microvesicles etc. Their dynamism and functional capabilities position them perfectly to regulate individual synapses and to be undoubtedly involved in optimizing information processing, learning and memory, and cognition.

## Author Contributions

IH and OP wrote the review.

## Conflict of Interest Statement

The authors declare that the research was conducted in the absence of any commercial or financial relationships that could be construed as a potential conflict of interest.

## References

[B1] Antonucci F. Turola E. Riganti L. Caleo M. Gabrielli M. Perrotta C.. (2012). Microvesicles released from microglia stimulate synaptic activity via enhanced sphingolipid metabolism. EMBO J. 31, 1231–1340. 10.1038/emboj.2011.489 22246184 PMC3297996

[B2] Bianco F. Pravettoni E. Colombo A. Schenk U. Möller T. Matteoli M.. (2005). Astrocyte-derived ATP induces vesicle shedding and IL-1 beta release from microglia. J. Immunol. 174, 7268–7277. 10.4049/jimmunol.174.11.7268 15905573

[B3] Burnstock G. (2008). Purinergic signalling and disorders of the central nervous system. Nat. Rev. Drug Discov. 7, 575–590. 10.1038/nrd2605 18591979

[B4] Chen T. Koga K. Li X.-Y. Zhuo M. (2010). Spinal microglial motility is independent of neuronal activity and plasticity in adult mice. Mol. Pain 6:19. 10.1186/1744-8069-6-19 20380706 PMC2857828

[B5] Davalos D. Grutzendler J. Yang G. Kim J. V. Zuo Y. Jung S.. (2005). ATP mediates rapid microglial response to local brain injury *in vivo*. Nat. Neurosci. 8, 752–758. 10.1038/nn1472 15895084

[B6] Dissing-Olesen L. LeDue J. M. Rungta R. L. Hefendehl J. K. Choi H. B. MacVicar B. A. (2014). Activation of neuronal NMDA receptors triggers transient ATP-mediated microglial process outgrowth. J. Neurosci. 34, 10511–10527. 10.1523/JNEUROSCI.0405-14.2014 25100586 PMC6802598

[B100] Dou Y. Wu H.-J. Li H.-Q. Qin S. Wang Y.-E. Li J.. (2012). Microglial migration mediated by ATP-induced ATP release from lysosomes. Cell Res. 22, 1022–1033. 10.1038/cr.2012.10 22231629 PMC3367529

[B7] Dunwiddie T. V. Diao L. Proctor W. R. (1997). Adenine nucleotides undergo rapid, quantitative conversion to adenosine in the extracellular space in rat hippocampus. J. Neurosci. 17, 7673–7682. 9315889 10.1523/JNEUROSCI.17-20-07673.1997 PMC6793930

[B8] Eyo U. B. Peng J. Swiatkowski P. Mukherjee A. Bispo A. Wu L.-J. (2014). Neuronal hyperactivity recruits microglial processes via neuronal NMDA receptors and microglial P2Y12 receptors after status epilepticus. J. Neurosci. 34, 10528–10540. 10.1523/JNEUROSCI.0416-14.2014 25100587 PMC4200107

[B9] Fontainhas A. M. Wang M. Liang K. J. Chen S. Mettu P. Damani M.. (2011). Microglial morphology and dynamic behavior is regulated by ionotropic glutamatergic and GABAergic neurotransmission. PLoS One 6:e15973. 10.1371/journal.pone.0015973 21283568 PMC3026789

[B10] Ginhoux F. Lim S. Hoeffel G. Low D. Huber T. (2013). Origin and differentiation of microglia. Front. Cell. Neurosci. 7:45. 10.3389/fncel.2013.00045 23616747 PMC3627983

[B24] Gomez-Nicola D. Perry V. H. (2015). Microglial dynamics and role in the healthy and diseased brain: a paradigm of functional plasticity. Neuroscientist 21, 169–184. 10.1177/1073858414530512 24722525 PMC4412879

[B11] Gyoneva S. Traynelis S. F. (2013). Norepinephrine modulates the motility of resting and activated microglia via different adrenergic receptors. J. Biol. Chem. 288, 15291–15302. 10.1074/jbc.M113.458901 23548902 PMC3663549

[B12] Hammarberg C. Schulte G. Fredholm B. B. (2003). Evidence for functional adenosine A3 receptors in microglia cells. J. Neurochem. 86, 1051–1054. 10.1046/j.1471-4159.2003.01919.x 12887702

[B13] Hanisch U.-K. Kettenmann H. (2007). Microglia: active sensor and versatile effector cells in the normal and pathologic brain. Nat. Neurosci. 10, 1387–1394. 10.1038/nn1997 17965659

[B14] Haynes S. E. Hollopeter G. Yang G. Kurpius D. Dailey M. E. Gan W.-B.. (2006). The P2Y12 receptor regulates microglial activation by extracellular nucleotides. Nat. Neurosci. 9, 1512–1519. 10.1038/nn1805 17115040

[B15] Hirasawa T. Ohsawa K. Imai Y. Ondo Y. Akazawa C. Uchino S.. (2005). Visualization of microglia in living tissues using Iba1-EGFP transgenic mice. J. Neurosci. Res. 81, 357–362. 10.1002/jnr.20480 15948177

[B16] Honda S. Sasaki Y. Ohsawa K. Imai Y. Nakamura Y. Inoue K.. (2001). Extracellular ATP or ADP induce chemotaxis of cultured microglia through Gi/o-coupled P2Y receptors. J. Neurosci. 21, 1975–1982. 11245682 10.1523/JNEUROSCI.21-06-01975.2001 PMC6762617

[B17] Hoshiko M. Arnoux I. Avignone E. Yamamoto N. Audinat E. (2012). Deficiency of the microglial receptor CX3CR1 impairs postnatal functional development of thalamocortical synapses in the barrel cortex. J. Neurosci. 32, 15106–15111. 10.1523/JNEUROSCI.1167-12.2012 23100431 PMC6704837

[B18] Hua Y. J. Smear M. C. Baier H. Smith S. J. (2005). Regulation of axon growth *in vivo* by activity-based competition. Nature 434, 1022–1026. 10.1038/nature03409 15846347

[B19] Jung S. Aliberti J. Graemmel P. Sunshine M. J. Kreutzberg G. W. Sher A.. (2000). Analysis of fractalkine receptor CX(3)CR1 function by targeted deletion and green fluorescent protein reporter gene insertion. Mol. Cell. Biol. 20, 4106–4114. 10.1128/mcb.20.11.4106-4114.2000 10805752 PMC85780

[B21] Kettenmann H. Hanisch U.-K. Noda M. Verkhratsky A. (2011). Physiology of microglia. Physiol. Rev. 91, 461–553. 10.1152/physrev.00011.2010 21527731

[B20] Kettenmann H. Kirchhoff F. Verkhratsky A. (2013). Microglia: new roles for the synaptic stripper. Neuron 77, 10–18. 10.1016/j.neuron.2012.12.023 23312512

[B22] Lee J. E. Liang K. J. Fariss R. N. Wong W. T. (2008). *Ex vivo* dynamic imaging of retinal microglia using time-lapse confocal microscopy. Invest. Ophthalmol. Vis. Sci. 49, 4169–4176. 10.1167/iovs.08-2076 18487378 PMC2652634

[B23] Li Y. Du X.-F. Liu C.-S. Wen Z.-L. Du J.-L. (2012). Reciprocal regulation between resting microglial dynamics and neuronal activity *in vivo*. Dev. Cell 23, 1189–1202. 10.1016/j.devcel.2012.10.027 23201120

[B25] Nimmerjahn A. Kirchhoff F. Helmchen F. (2005). Resting microglial cells are highly dynamic surveillants of brain parenchyma *in vivo*. Science 308, 1314–1318. 10.1126/science.1110647 15831717

[B26] Ohsawa K. Sanagi T. Nakamura Y. Suzuki E. Inoue K. Kohsaka S. (2012). Adenosine A3 receptor is involved in ADP-induced microglial process extension and migration. J. Neurochem. 121, 217–227. 10.1111/j.1471-4159.2012.07693.x 22335470

[B27] Oliveira J. F. Sardinha V. M. Guerra-Gomes S. Araque A. Sousa N. (2015). Do stars govern our actions? Astrocyte involvement in rodent behavior. Trends Neurosci. 38, 535–549. 10.1016/j.tins.2015.07.006 26316036

[B28] Orr A. G. Orr A. L. Li X.-J. Gross R. E. Traynelis S. F. (2009). Adenosine A(2A) receptor mediates microglial process retraction. Nat. Neurosci. 12, 872–878. 10.1038/nn.2341 19525944 PMC2712729

[B29] Paolicelli R. C. Bolasco G. Pagani F. Maggi L. Scianni M. Panzanelli P.. (2011). Synaptic pruning by microglia is necessary for normal brain development. Science 333, 1456–1458. 10.1126/science.1202529 21778362

[B30] Parkhurst C. N. Yang G. Ninan I. Savas J. N. Yates J. R. Lafaille J. J.. (2013). Microglia promote learning-dependent synapse formation through brain-derived neurotrophic factor. Cell 155, 1596–1609. 10.1016/j.cell.2013.11.030 24360280 PMC4033691

[B31] Pascual O. Ben Achour S. Rostaing P. Triller A. Bessis A. (2012). Microglia activation triggers astrocyte-mediated modulation of excitatory neurotransmission. Proc. Natl. Acad. Sci. U S A 109, E197–E205. 10.1073/pnas.1111098109 22167804 PMC3268269

[B32] Penuela S. Gehi R. Laird D. W. (2013). The biochemistry and function of pannexin channels. Biochim. Biophys. Acta 1828, 15–22. 10.1016/j.bbamem.2012.01.017 22305965

[B33] Perea G. Sur M. Araque A. (2014). Neuron-glia networks: integral gear of brain function. Front. Cell. Neurosci. 8:378. 10.3389/fncel.2014.00378 25414643 PMC4222327

[B34] Peri F. Nüsslein-Volhard C. (2008). Live imaging of neuronal degradation by microglia reveals a role for v0-ATPase a1 in phagosomal fusion *in vivo*. Cell 133, 916–927. 10.1016/j.cell.2008.04.037 18510934

[B35] Prinz M. Priller J. (2014). Microglia and brain macrophages in the molecular age: from origin to neuropsychiatric disease. Nat. Rev. Neurosci. 15, 300–312. 10.1038/nrn3722 24713688

[B36] Ransohoff R. M. Perry V. H. (2009). Microglial physiology: unique stimuli, specialized responses. Annu. Rev. Immunol. 27, 119–145. 10.1146/annurev.immunol.021908.132528 19302036

[B37] Schafer D. P. Lehrman E. K. Kautzman A. G. Koyama R. Mardinly A. R. Yamasaki R.. (2012). Microglia sculpt postnatal neural circuits in an activity and complement-dependent manner. Neuron 74, 691–705. 10.1016/j.neuron.2012.03.026 22632727 PMC3528177

[B38] Scianni M. Antonilli L. Chece G. Cristalli G. Di Castro M. A. Limatola C.. (2013). Fractalkine (CX3CL1) enhances hippocampal N-methyl-D-aspartate receptor (NMDAR) function via D-serine and adenosine receptor type A2 (A2AR) activity. J. Neuroinflammation 10:108. 10.1186/1742-2094-10-108 23981568 PMC3765929

[B39] Tremblay M. E. Lowery R. L. Majewska A. K. (2010). Microglial interactions with synapses are modulated by visual experience. PLoS Biol. 8:e1000527. 10.1371/journal.pbio.1000527 21072242 PMC2970556

[B40] Wake H. Moorhouse A. J. Jinno S. Kohsaka S. Nabekura J. (2009). Resting microglia directly monitor the functional state of synapses *in vivo* and determine the fate of ischemic terminals. J. Neurosci. 29, 3974–3980. 10.1523/JNEUROSCI.4363-08.2009 19339593 PMC6665392

[B41] Wu L.-J. Zhuo M. (2008). Resting microglial motility is independent of synaptic plasticity in mammalian brain. J. Neurophysiol. 99, 2026–2032. 10.1152/jn.01210.2007 18256162

